# Investigation and impact of mammalian adaptation markers on H5N8 high pathogenicity avian influenza polymerase activity

**DOI:** 10.1038/s44298-026-00188-3

**Published:** 2026-04-09

**Authors:** Maxime Fusade-Boyer, Arthur Kocher, Pierre Bessière, Thomas Figueroa, Charlotte Foret-Lucas, Timothée Vergne, Christophe Chevalier, Mariette F. Ducatez, Romain Volmer

**Affiliations:** 1 Univ Toulouse, ENVT, INRAE, IHAP, Toulouse, France; 2 UMR892 VIM, UVSQ, INRAE, Université Paris-Saclay, Jouy-en-Josas, F78352, France

**Keywords:** Genetics, Microbiology, Molecular biology

## Abstract

Highly pathogenic H5Nx viruses of clade 2.3.4.4b have spread worldwide, causing major economic losses and increased human exposure. Since 2020, multiple mammalian infections have been reported, raising concerns about further adaptation to mammalian hosts. We analyzed influenza A virus sequences from the Influenza Virus Database at the National Center for Biotechnology Information to identify new mammalian adaptation markers in the polymerase complex and nucleoprotein, using recursive partitioning. These markers were grouped into “proteotypes” to assess their co-occurrence and association with host origin. This analysis revealed distinct groups of proteotypes linked to mammalian adaptation, including those seen in historical and pandemic human strains. Identified mutations were introduced alone or in combination into a 2.3.4.4b H5N8 virus to evaluate their impact on polymerase activity in mammalian cells using a minigenome assay. PB1 V336I and PB2 K702R increased polymerase activity in human cells, particularly with PB2 E627K, supporting enhanced surveillance of 2.3.4.4b H5Nx viruses. These findings highlight mutation combinations relevant for enhanced surveillance of 2.3.4.4b H5Nx viruses.

## Introduction

Highly pathogenic avian influenza viruses (HPAIV) have dramatically expanded around the world over recent years^1^. In particular, since 2016, clade 2.3.4.4b H5N8 viruses spread globally in many countries in Europe, Africa, the Middle East, Asia, and were responsible for high mortality in various species of wild and domestic birds^2–6^. This subtype’s sustained circulation and ability to evolve by reassortment led to the emergence of different H5Nx subtypes. Among these, H5N1 is currently the most prevalent worldwide^7^, and was recently found responsible for numerous mammalian infections and several human cases^8–11^. Human infections with some HPAIV are associated with considerable fatality rates, ranking between 39 and 59% ^12^. However, although these viruses are able to infect mammals, they are not yet fully human-adapted and are unable to transmit efficiently within the human population. Efficient human-to-human transmission capacity can be acquired by avian influenza viruses through adaptive mutations in different viral genes or through reassortment, which has resulted in repeated human flu pandemics in the past^13^. In this context, it is crucial to better understand the molecular determinants of host adaptation in avian influenza A viruses (IAV) to improve risk assessment of currently circulating strains and adapt mitigation strategies.

The viral polymerase complex, composed of polymerase basic 1 (PB1), polymerase basic 2 (PB2) and polymerase acidic (PA), and the nucleoprotein (NP) are an important determinant of host adaptation. In particular, mutations in these genes can affect the optimal temperature for viral replication, which is a key parameter of host range restriction. Indeed, avian-adapted influenza viruses usually replicate efficiently around 41 °C, which is much higher than the temperature of ~33 °C found in the human upper respiratory tract. Consequently, avian influenza virus strains acquiring the ability to replicate around 33 °C would be better equipped to replicate in the human upper respiratory tract, one of the prerequisites for efficient human-to-human transmission^14^. In addition to facilitating protein interactions between the polymerase complex and host proteins belonging to the ANP32 family, the amino acid substitution PB2 E627K is known to increase polymerase activity at 33 °C as well as viral replication, pathogenicity and transmission of different subtypes of avian influenza viruses in mammals. To date, it is the best-documented adaptive mutation located in the influenza virus polymerase complex^14–22^. The PB2 E627K mutation is not the only marker associated with human adaptation; other mutations, such as PB2 T271A, PB2 D701N, and many others have also been described as facilitating adaptation to mammalian hosts, with new mammalian adaptation mutations being regularly identified^23^. Consequently, identifying new markers of mammalian adaptation is crucial to improving risk analyses and detecting strains of IAV better adapted to mammals at an earlier stage. One difficulty in this regard is that such markers are not necessarily associated with strict host ranges and may be present in both avian and mammalian-associated strains. In addition, the combination of several mutations affecting different loci can be necessary to cause biological effects. Here, we analyzed an extensive set of IAV sequences associated with host species information using recursive partitioning to identify combinations of mammalian adaptation markers in the polymerase complex. We then experimentally measured the impact of some of the identified mutations, which appeared the most likely to be involved in early stages of adaptation to mammalian hosts, alone or in association, on the polymerase activity of clade 2.3.4.4b H5N8 virus, using a minigenome assay in mammalian cells.

## Results

### Bioinformatic identification of mammalian adaptation markers in the polymerase complex of IAVs

**Fig. 1:**
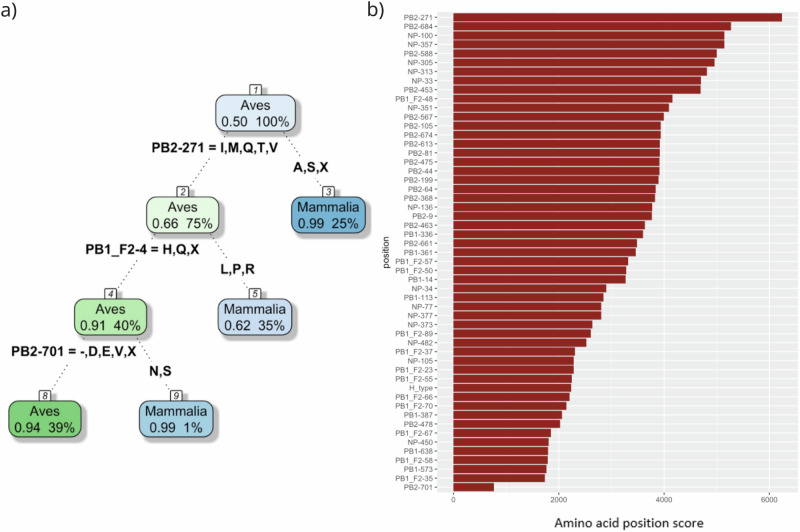
Discriminant amino acid position identified by recursive partitioning. **a** Decision tree estimated using recursive partitioning for the prediction of host class based on PB1, PB1-F2, PB2 and NP amino acid sequences. At each node, the primary split (i.e. most discriminating AA position) is indicated, together with avian or mammalian-like residues (on the left and right branches of the split, respectively). **b** Importance score of amino acid position identified as primary or surrogate splits through recursive partitioning, from the highest (at the top) to the lower (at the bottom). Position PB2-271 is identified as the most discriminating between avian and mammalian viruses.

**Fig. 2:**
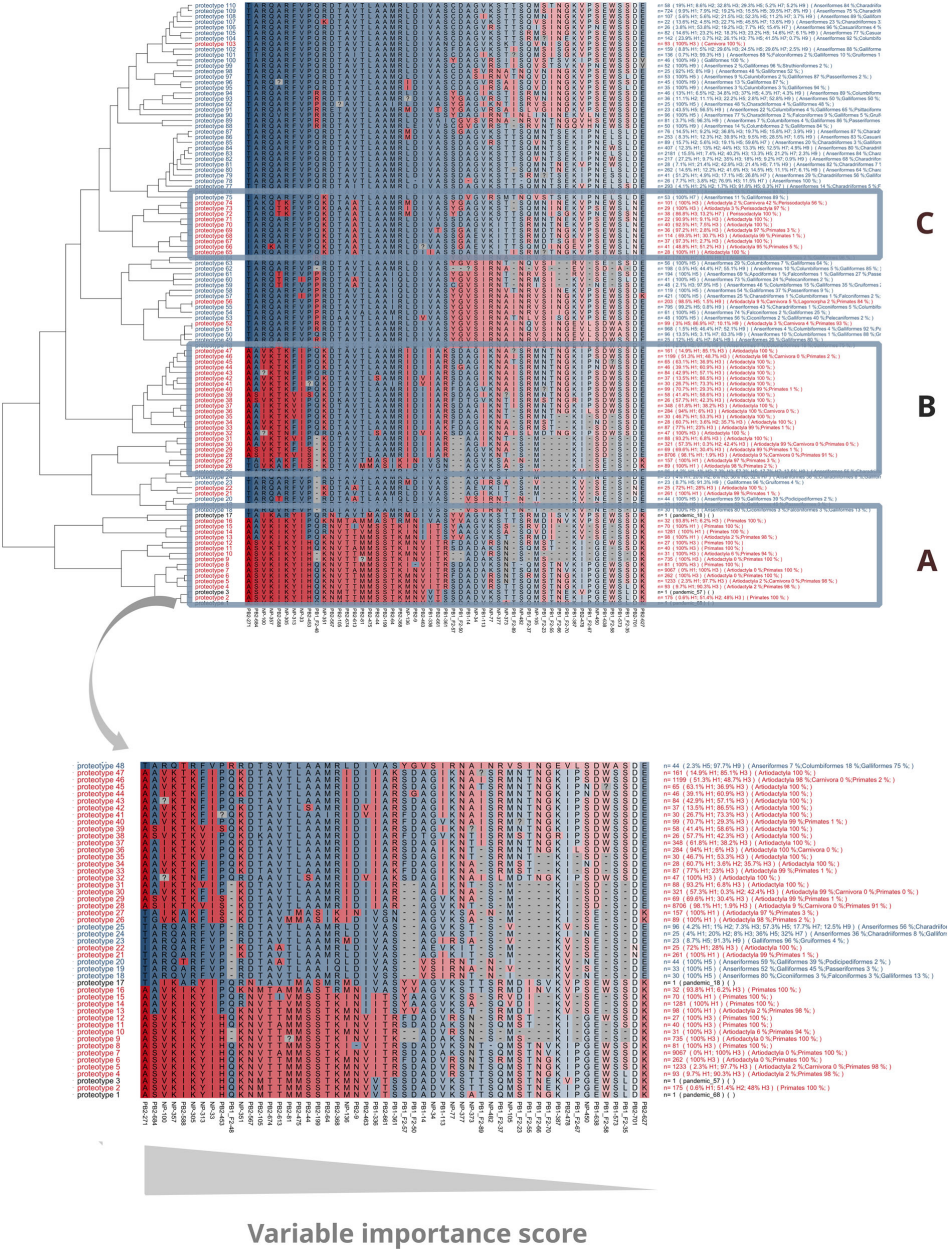
Combinations of amino acid residues (proteotypes) observed at discriminating positions across mammalian and avian influenza A strains, as identified through recursive partitioning. Proteotypes written in red and blue correspond to strains detected in mammals and birds, respectively. Those written in black correspond to pandemic strains from 1918, 1957 and 1968. In the alignment, amino acid positions were ordered according to their discriminating power with respect to host class, as measured by the importance score. Amino acid residues were colored according to their main host association (red or blue if identified as mammalian or avian-like, respectively), with a level of transparency proportional to their importance score. The number of strains exhibiting each proteotype and their distribution across H subtypes and host taxa are reported on the right of the plot. **A** Proteotypes of human seasonal influenza viruses very well adapted to human. **B**, **C** Viruses adapted to mammals, mainly isolated from swine.

The recursive partitioning analysis resulted in a decision tree including three splits (Fig. 1a). 52 discriminating AA positions located in PB2, PB1, NP and PB1-F2 were identified, with importance scores ranging from 767 to 6235 (Fig. 1b). The higher the importance score, the more the identified mutations are specifically associated with influenza viruses detected in mammalian hosts and may, therefore, confer a selective advantage in mammals. While some of the mutations identified, such as PB2 T271A, D701N, A684S or A588I/T, have already been well described, others have been poorly or even not described in the literature. These include several mutations of PB1 (V14A, K387E, E638D, S573A), NP (R77K, S34E/G/D, L136I, T373A/N, N377S, N482S, N450P) and almost all mutations identified in the PB1-F2 protein. No discriminant mutations were identified in the PA segment, but this could stem from the fact that much less data was available for this segment compared to the other segments considered in our analysis. All the positions identified by our analysis are listed in Table 1, specifying the amino acids identified as more frequent in mammalian or avian hosts. The results were then plotted to show the different combination of AA residues at these discriminating positions, referred to as proteotypes hereafter, occurring in IAV strains represented in our datasets in order to highlight potential mutation associations (Fig. 2). Three main groups were defined, based on proteotypes associated with viral sequences from infected mammals. Group A included proteotypes of H1, H2 and H3 subtypes corresponding to viruses responsible for human seasonal flu and for the 1918, 1957 and 1968 pandemics. Most of these human-adapted and highly transmissible viruses harbored about 50% of the most discriminant adaptive mutations identified in the computational analysis (Fig. 2). Groups B and C proteotypes corresponded to mammalian viruses, mainly detected in swine, which had less mammalian-like mutations compared to those from group A, but that were still able to infect mammals. Group B also included the proteotype of the 2009 H1N1 pandemic virus, which had inherited the PB2 and PA segments from an avian influenza virus, the PB1 segment from a human virus and the NP segment from a swine virus^24^. Of note, the same associations of mammalian markers were identified in different proteotypes, suggesting that the combination of these mutations might play an important role in the adaptation of the virus to mammalian hosts.

**Table 1:** List of 52 amino acid positions identified as discriminant between avian and mammalian hosts by using recursive portioning analyses

Protein	Amino acid position	Avian	Mammalian	Protein	Amino acid position	Avian	Mammalian
PB2	9	D	N	PB1-F2	23	N/D	S/G
	44	A	S		35	L	S
	64	M/I	T		37	Q	L/R
	81	T	M/V		48	Q	P/R
	105	A/T	M/V		50	A/D	V/G
	199	A	S		55	T	I
	271	T	A		57	F/S/C	Y
	368	Q/R	K		58	L	W
	453	P	S/H		66	S	N
	463	I	V		67	P	L/H
	475	L	M		70	V/E	G/D
	478	I	V		89	T	I
	567	E/D	N	NP	33	V	I
	588	A	I/T		34	A/G/D/E	S
	613	I/V	T/A		77	K	R
	661	A/S/G/V	T		100	R	I/V
	674	A	T		105	M/I	
	684	A/G	S		136	L	I/M
	701	D	N		305	R	K/N
PB1	14	A	V		313	F	Y/V
	113	A/V	I		351	R	K
	336	V	I		357	Q	K
	361	S/N	R/K		373	T/K	S/A
	387	K	E/Q/R		377	I/S	N
	573	S	A		450	N/G	S
	638	E	D		482	S	N

### Mammalian-adaptation mutations in the PB2 and PB1 genes of H5N8 virus of clade 2.3.4.4b enhance polymerase activity in minigenome assays when they are associated with PB2 E627K

The above-mentioned bioinformatic analyses were used to identify the spectrum and combinations of mutations discriminating strains associated with avian or mammalian hosts. We selected mutations which were (i) highly discriminant (variable importance score >3600) and (ii) found in proteotypes associated with mammal-infecting strains which carried a little number of mammal-like mutations. The rationale behind this was to select mutations potentially involved in the earliest stages of adaptation. Indeed, some mutations identified in viruses already well adapted to mammals may have little or no detectable impact when introduced alone in an avian genetic background, as they could result from a later-stage emergence to stabilize or optimize pre-existing early-stage mammalian adaptation mutations. We then checked that the selected mutations had already been reported on avian H5Nx viruses, to ensure that they were viable on an avian H5N8 virus. Therefore, the mutations PB2 V613A, PB1 V336I and NP R351K were selected for polymerase activity assays. These mutations were first tested for polymerase activity in swine cells, as they were identified in swine-infecting strains that showed few mammal-like mutations otherwise. Polymerase activity was also measured in human HEK-293T cells. The PB2 E627K, D701N and K702R mutations, although identified as poorly discriminant or not detected by our analysis, were nevertheless included due to their well-documented roles in mammalian adaptation and because they have already been reported in clade 2.3.4.4b H5Nx viruses, thereby providing a genetic background more representative of field strains for risk assessment purposes (Briand et al.^25^). These mutations were used to provide a permissive context to evaluate whether mutations identified as potential early-stage adaptation markers could exert measurable effects on polymerase activity in mammalian cells, either alone or through synergistic interactions. Polymerase activity in human HEK-293T and swine NPTr cells was assessed at 37 °C and 33 °C, the swine and human upper respiratory tract temperatures, respectively.

**Fig. 3:**
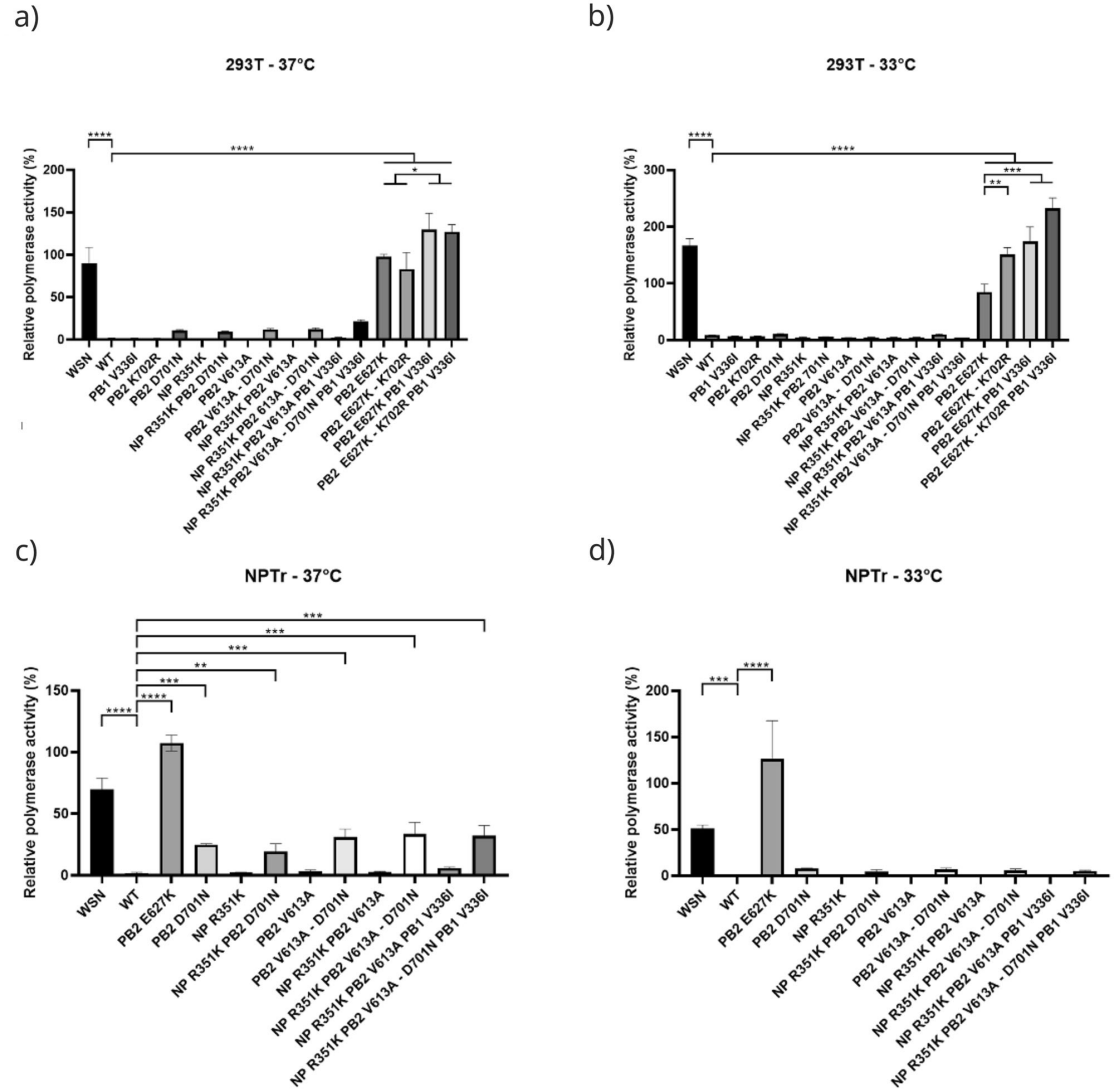
Polymerase activity of RNPs from H5N8 wild-type and mutant viruses in human and swine cells. Measures of polymerase activity were performed using a minigenome assay in HEK-293T and NPTr cells 24 h post-transfection. The virus A/WSN/1933 H1N1 (WSN), well adapted to the human host was used as positive control. All other mutations and combination of mutations were performed on ribonucleoproteins (RNPs) from H5N8 virus. Renilla luciferase was used as internal control to normalize Firefly luciferase activity. Polymerase activity of selected mutations performed in HEK-293T cells at 37 °C (**a**) and at 33 °C (**b**). Polymerase activity of selected mutations performed in NPTr cells at 37 °C (**c**) and at 33 °C (**d**). The error bars show the mean with SEM from at least two experiments carried out in triplicate and one-way ANOVA was performed for statistical analysis. *P < 0.05; **P < 0.01; ***P < 0.001; ***P < 0.0001.

In both swine and human cells, the wildtype H5N8 virus showed a very low level of polymerase activity in comparison with the human-adapted strain A/WSN/1933 H1N1 (WSN) used as control, supporting the low mammalian adaptation of H5N8 wildtype virus polymerase (Fig. 3). As expected, the mutation PB2 E627K significantly increased polymerase activity at 37 °C and 33 °C in both cell types (with a >80% increase), but surprisingly, PB2 D701N had little impact on polymerase activity. Indeed, this mutation did not induce a significant increase in human cells and only a slight increase of the polymerase activity in swine cells at 37 °C. The combination NP R351K, PB1 V336I and PB2 V613A with PB2 D701N did not lead to a significant increase of polymerase activity in HEK-293T and NPTr cells. At both temperatures, the level of polymerase activity was not significantly higher than the increase due to the mutation D701N of PB2 alone. Although not statistically significant, a 5.6-fold increase in polymerase activity was observed with PB2 D701N in HEK-293T cells at 37 °C compared with the wild-type polymerase. The combination of PB2 D701N with NP R351K, PB1 V336I, and PB2 V613A resulted in an ~11.6-fold increase relative to the wild type, suggesting a possible cooperative effect of these mutations in the presence of PB2 D701N. In NPTr cells at 37 °C, when excluding the major mammalian-adaptation markers PB2 D701N and PB2 E627K, the highest increase in polymerase activity was a 3.2-fold change relative to the wild-type polymerase, observed with the combined mutations NP R351K, PB1 V336I, and PB2 V613A. When combined with PB2 E627K, mutations PB1 V336I or PB2 K702R significantly increased polymerase activity compared to PB2 E627K alone in HEK-293T cells at 33 °C. The effect was even stronger when PB1 V336I and PB2 K702R were both combined with PB2 E627K, with a significant increase. However, these mutations alone did not cause any significant change in polymerase activity in the absence of PB2 E627K.

## Discussion

Since their global spread in 2016, 2.3.4.4b HPAI H5Nx viruses have greatly impacted poultry industry in several countries and have led to high mortality in various species of wild and domestic birds^3,26^. The detection of H5N8 and other H5Nx viruses in several mammalian species and more recently in cattle and humans, revived the concerns posed by these viruses for public health^8,11,27^. Although most of the 2.3.4.4b clade H5Nx remain poorly adapted to mammals, several mammalian-adaptation mutations previously described in the literature were found in isolates collected from different hosts. This is for example the case with the mutations PB2 E627K and PB2 T271A, which have been found in several isolates of clade 2.3.4.4b H5Nx collected recently in different mammalian species, and as well as in birds in the case of PB2 E627K^25,28,29^. The detection of PB2 E627K in birds shows that certain mammalian-adapting mutations can already be present while the virus is circulating in the avian compartment, before their transmission to mammals where they can be selected^29–31^. While a combination of different mutations can be required for successful adaptation and changes in phenotype, in some cases, one mutation alone can be sufficient to dramatically change the pathogenicity of the virus in mammals, as it was shown for PB2 E627K in H7N7 virus^32^. In this context, the surveillance of circulating strains coupled to the investigation of mutations responsible for changes in virus phenotype or host adaptation are critical in pandemic preparedness. In the present study, we first performed a computational analysis to identify molecular markers of adaptation to mammalian hosts in influenza A viruses. While many discriminant markers found in our analysis had already been reported in the literature, others were poorly or not described, such as NP L136I or PB2 P453S. These were usually the least discriminant between avian and mammalian viruses, suggesting a limited role in host adaptation. Surprisingly, some well-described mammalian-adaptation markers such as PB2 E627K were not identified by our analysis. This can be explained by their heterogeneous distribution across host species, with PB2 E627K being prevalent in certain avian lineages, such as clade 2.2 H5N1 viruses^33^ while remaining relatively uncommon in human 2009 H1N1 pandemic and Eurasian swine H1N1 and H3N2 swine triple reassortant internal genes (TRIG) influenza viruses^34,35^. As a consequence, its discriminative power is reduced in large, heterogeneous datasets where host origin does not strictly reflect the stage of host adaptation. In addition, some adaptive mutations may require prolonged circulation in mammalian hosts to become fixed and may therefore be underrepresented or absent in datasets dominated by sporadic spillover events or early-stage mammalian infections^36^. The effect of these mutations was tested alone or in combination in order to identify potential synergies. They were tested in association with PB2 E627K, K702R and D701N, three well known mammalian-adaptive markers which had already been reported in some strains of H5Nx clade 2.3.4.4b. In swine cells, PB2 E627K was used solely as a positive control, whereas PB2 D701N was selected as the canonical mammalian-adaptation mutation due to its higher frequency in swine-adapted viruses compared to PB2 E627K or K702R^37^. Our results from minigenome assays showed the low level of adaptation of H5N8 virus polymerase complex in human and swine cells and confirmed the effect of PB2 E627K on polymerase activity, underlining its role as an enhancer of other adaptive mutations. Indeed, none of the tested mutations increased the polymerase activity significantly at 33 °C or 37 °C when introduced alone. However, when combined with PB2 E627K, both PB1 V336I and PB2 K702R increased the polymerase activity of the H5N8 virus at 33 °C in human cells. In addition, the combination of PB2 E627K with PB1 V336I also enhanced polymerase activity at 37 °C. This increase was higher when PB1 V336I and PB2 K702R were both introduced in combination with PB2 E627K. These observations suggest that PB1 V336I and PB2 K702R may act as permissive or potentiating mutations of PB2 E627K. PB2 E627K is known to stabilize polymerase function in mammalian cells, notably through improved interactions with host factors, as previously demonstrated for ANP32 proteins, and through an overall increase in polymerase activity^38^. This synergistic effect observed when PB1 V336I and PB2 K702R were combined with PB2 E627K could implicate modifications of intersubunit interactions within the polymerase complex when PB2 E627K is present. In this context, secondary substitutions such as PB1 V336I, which is located within a region implicated in cRNA binding, may modulate polymerase–RNA interactions or template engagement, thereby enhancing polymerase efficiency once a permissive background has been established by PB2 E627K^39^. PB2 K702R, located in the vicinity of the PB2 627 domain, could further contribute by modulating conformational flexibility or host-factor engagement. The mutation PB1 V336I has been identified by several computational studies as a mammalian adaptation marker, but its biological effect on viral replication and polymerase activity had never been reported to our knowledge. The fact that this increase in polymerase activity was observed at 33 °C, the temperature of the human upper respiratory tract, suggests that these combinations of mutations could favor virus replication in the upper respiratory tract, one of the prerequisites for efficient human-to-human transmission. A significant increase in polymerase activity was also observed with the PB2 D701N mutation, but only in swine cells and at 37 °C. These results can be explained by the fact that the H5N8 wild-type polymerase exhibits very low baseline activity in HEK293T cells at both 33 °C and 37 °C, indicating that this avian backbone is strongly restricted in human and swine cells. Given that the impact of the PB2 D701N mutation can depend on the genetic background, this suggests that mutations such as PB2 D701N may require a more permissive genetic context to exert measurable effects in HEK293T cells^40^. Nevertheless, the increase of polymerase activity observed within the same viral genetic background in NPTr cells suggests the involvement of host factors. PB2 D701N is known to enhance interactions with cellular importins, whose expression levels or binding affinities may differ between porcine and human cells. The relatively well-described PB2 D701N mutation is a mutation that appears to be selected more in swine than in human which is consistent with the results observed and supports the role of host-factor–mediated restriction^41^.

No increase was observed with PB2 K702R either at 37 or 33 °C. This observation is consistent with the variable results reported in the literature concerning PB2 K702R, a mutation of which the impact on polymerase activity seems highly dependent on the genetic background of the virus. Previous experiments showed no effect of PB2 K702R on the polymerase activity of an Asian avian H5N1 virus either at 37 °C or 33 °C, while another study showed a significant increase of polymerase activity following the introduction of this mutation in a European avian H5N1 virus^42,43^. The mutations PB2 V613A, NP R351K and PB1 V336I did not lead to an increase of polymerase activity even in NPTr cells, associated or not with PB2 D701N, suggesting the requirement of other mutations than the tested combination in H5N8 virus to increase polymerase activity. One should however keep in mind that an increase in viral replication without increased polymerase activity can sometimes occur^43^. This highlights one of the limitations of our experimental setting, which focuses solely on polymerase activity, to avoid potential hazards associated with gain-of-function studies with live viruses, making it blind to other mechanisms through which certain mutations could affect the viral cycle. Though the polymerase complex and NP are an important target in mammalian adaptation, careful consideration should also be given to other gene segments. The affinity for α2-6 sialic acid receptors and a low pH fusion of the HA are critical parameters in human adaptation^44^, which have not been acquired completely yet by H5Nx virus of clade 2.3.4.4b^11^. Other viral proteins have also been shown to be involved in host adaptation of AIV such as NA, M1 and M2, NS1 and NS2^23^. Regarding the temperatures tested in the minigenome assays, although experiments at 40 °C were not performed in this study, assessing polymerase activity at this temperature could provide additional information regarding temperature-dependent effects, particularly in relation to avian body temperature and febrile conditions in human. The article recently published by Turnbull et al. particularly highlights this aspect, showing that fever can create a replicative environment favorable to avian viruses in a mammalian host, whose physiological body temperature is lower than that of birds, without necessarily relying on adaptive mutations^45^. Indeed, the phenotypic characteristics of avian viruses that confer high polymerase activity at the elevated body temperature of birds may become advantageous in a febrile environment, compared with seasonal influenza viruses, which are impaired by this increase in temperature. Nevertheless, in the context of clade 2.3.4.4b zoonotic viruses, excepting Asian H5N6 viruses, we note that human infections associated with this clade have been characterized by asymptomatic, mild, or atypical clinical presentations, including conjunctival involvement and the absence of marked febrile symptoms in a proportion of cases. In this context, the evaluation of polymerase activity at temperatures representative of the mammalian respiratory tract (33 °C and 37 °C) was prioritized in the present study. In addition, fever typically develops 2–5 days, up to 7, after zoonotic H5N1 infection, implying that efficient viral replication at physiological mammalian temperatures, particularly around 37 °C in case of the lower respiratory tract infection, is likely required before exposure to higher febrile temperatures^46^. Therefore, assessing replication competence at 37 °C remains a primary and biologically relevant condition.

By identifying mutations co-selected and specifically associated with specific host species, our work provides a new methodological approach to identify combinations of mammalian-adaptation mutations, which could be considered to better understand the different evolutionary pathways leading to mammalian adaptation of avian influenza viruses. Our results highlight several mutations which could be involved in adaptation of avian influenza A viruses to mammalian hosts. More specifically, we showed that the mutations PB2 K702R and PB1 V336I can act synergistically with PB2 E627K to increase the polymerase activity of HPAI H5N8 virus of clade 2.3.4.4b in human cells. These associations of amino acids should therefore be monitored in 2.3.4.4b clade H5Nx viruses for public health risk analysis.

## Methods

### IAV sequence dataset and alignment

All sequences of the IAV segments encoding PB2, PB1, PA and NP available in the NIAID Influenza Research Database (IRD) were downloaded in July 2018. Duplicated sequences, or sequences for which subtype or host species information was missing were excluded. Host species information was carefully curated and strains were classified as either avian or mammalian-associated. We then used the *transeq* tool from the EMBOSS package^47^ to translate all DNA sequences. Resulting amino acid sequences were kept only if their length was at least 90% of the expected complete protein length^48^, and if they contained no more than 10 ambiguous characters. PB1-F2 AA sequences were obtained by translating segment 2 (PB1) from the alternative starting codon at position 95. All AA sequences were aligned using *MAFFT v. 7*^49^. Sequences exhibiting unique indels that were not shared with any other sequence were excluded. The final dataset contained a total of 186,130 amino acid sequences including ~44k sequences for each of the investigated proteins (including PB1-F2), except PA for which only ~10k sequences were retrieved. These sequences belonged to a total of 45,049 strains encompassing 123 IAV subtypes. 63.3% and 37,7% of these strains were isolated from mammals and birds, respectively. Mammalian strains were mostly isolated from humans (79.1%) and pigs (19.5%). Mallard ducks and chickens were the most frequent avian hosts (37.7% and 20.9%, respectively).

### Recursive partitioning analysis

The curated amino acid sequence dataset was used for a recursive partitioning analysis^50^ with the R package *rpart* to identify host-specific amino acid residues in the investigated proteins. Recursive partitioning provides a decision tree aiming at classifying observations into subgroups using iterative splits (decisions) based on a set of selected predictive variables. Here, the host class (mammalian or avian) was used as the response variable and all variable amino acid positions of each studied protein were used as predictive variables. To reduce computation time, highly conserved amino acid positions that were identical in more than 99% of the sequences were excluded. The HA subtype was also added as a predictive variable to identify potential subtype-specific markers of adaptation. Only the HA subtypes comprising at least 50 mammalian and avian strains were retained for the analysis (H1, H2, H3, H5, H7 and H9). Furthermore, each observation was weighted by the inverse of corresponding host*subtype counts in the dataset to compensate for lineage-specific sampling biases. The “class” method was used with a minimum complexity parameter of 2% and a 100-fold cross-validation. All other parameters were set to default.

### Identification and selection of host adaptation markers

All amino acid positions retained in the decision tree generated through recursive partitioning were considered as potential host-adaptation markers. These included (i) “primary splits”, i.e. the most discriminant amino acid position at a given node of the tree, as well as (ii) “surrogate splits”, i.e. discriminant amino acid positions correlating with the primary split at a given node. Only surrogate splits showing at least 40% agreement with the primary split (as measured by the corrected agreement score), and a maximum of 25 surrogate splits per node were retained. The discriminative power of each retained amino acid position was measured using the “variable importance score”.

We then investigated potential associations between mutations involved in host adaptation across IAV lineages. To this end, we identified all combinations of AA residues at identified discriminating positions (referred to as proteotypes hereafter) in our dataset. For visualization purposes, we grouped proteotypes based on (i) the host class in which they were found and (ii) hierarchical clustering based on the raw similarity between proteotype sequences, with a clustering threshold of 20%. Proteotypes groups found in less than 20 IAV strains were excluded. For each remaining proteotype group, we generated a consensus amino acid sequence using a 50% majority rule. Proteotypes associated with pandemic strains (A/Brevig_Mission/1/1918, A/Shanghai/202/1957 and A/Hong_Kong/1/1968) were identified and plotted separately.

### Minigenome assays

The reverse genetic plasmid encoding the wild-type or mutated genes PB2, PB1, PA and NP of the A/duck/France/161108h/2016 (H5N8) were modified by mutagenesis using the kit In-Fusion HD Cloning Plus (Takara Bio) to delete the RNA polymerase I promotor, not necessary for polymerase activity assays, before to be used in minigenome assay. This strain was used as a representative clade 2.3.4.4b H5N8 virus with a predominantly avian genetic background and without major canonical mammalian adaptation markers in the polymerase complex. Human embryonic kidney (HEK-293T) cells and newborn pig trachea (NPTr) cells cultured in 12-well plates were transfected in Opti-MEM medium with 100 µg/ml penicillin/streptomycin, using the lipophilic transfection LTX with Plus reagent (Invitrogen, Thermo Fisher Scientific Inc.) with 0.25 μg of pHW2000-PB2, pHW2000-PB1, pHW2000-PA, pHW2000-NP (wild-type or muted), 0.25 µg of pPolI-Firefly plasmid encoding the Firefly luciferase gene in negative polarity flanked by the 5’ and 3’ non-coding regions (NCR) of NS segment of AIV and 0.25 µg of internal control plasmid expressing Renilla luciferase (Promega) too^34,51,52^. Cells were incubated at 33 °C or 37 °C with 5% CO_2_. Twenty-four hours after transfection, cells were harvested and lysed using a Dual-Luciferase Reporter Assay System (Promega). Firefly and Renilla luciferase activity were measured using a plate reader (CLARIOstar, BMG Labtech). Firefly values were normalized with the Renilla values from the internal control plasmid. Two positive controls were used in the minigenome assays: the polymerase complex of A/WSN/1933 (H1N1), a human-adapted strain, and the H5N8 polymerase complex carrying the PB2 E627K mutation, representing the introduction of a canonical mammalian-adaptation marker into an avian backbone. Polymerase activity was normalized to the H5N8 PB2 E627K condition, which was set as 100%.

## Data Availability

All sequences used for this analysis are publicly available on NCBI and the data that support the findings of this study are available from the corresponding author, upon reasonable request.
